# Exercise and behaviour change support for individuals living with and beyond cancer: Interim results and program satisfaction of the EXCEL study

**DOI:** 10.1016/j.jsampl.2024.100055

**Published:** 2024-02-24

**Authors:** Chad W. Wagoner, Julianna Dreger, Melanie R. Keats, Margaret L. McNeely, Colleen Cuthbert, Lauren C. Capozzi, George J. Francis, Linda Trinh, Kristin Campbell, Daniel Sibley, Jodi Langley, S. Nicole Culos-Reed

**Affiliations:** a Faculty of Kinesiology, University of Calgary, Canada; b Ohlson Research Initiative, Arnie Charbonneau Research Institute, Cumming School of Medicine, University of Calgary, Canada; c Department of Community Health Sciences, Cumming School of Medicine, University of Calgary, Calgary, Canada; d Faculty of Health, School of Health and Human Performance, Dalhousie University, Canada; e Department of Medicine, Division of Medical Oncology, Nova Scotia Health, Canada; f University of Alberta, Canada; g Supportive Care Services, Cancer Care Alberta, Canada; h Faculty of Nursing, University of Calgary, Canada; i Physical Medicine & Rehabilitation, Department of Clinical Neurosciences, Cumming School of Medicine, University of Calgary, Canada; j Faculty of Kinesiology and Physical Education, University of Toronto, Canada; k Physical Therapy, Faculty of Medicine, University of British Columbia, Canada; l Department of Oncology, Cumming School of Medicine, University of Calgary, Canada; m Department of Psychosocial Resources, Tom Baker Cancer Centre, Alberta Health Services, Canada

**Keywords:** Physical activity, Patient-reported outcomes, Physical function, Online exercise, Program satisfaction, Preliminary effectiveness

## Abstract

**Purpose:**

Examine the impact of the EXercise for Cancer to Enhance Living Well's (EXCEL) 10–12-week exercise and behaviour change support intervention on secondary effectiveness outcomes, including patient-reported outcomes, physical function, and program satisfaction.

**Methods:**

Individuals with cancer up to 3 years post treatment with any tumour type were eligible. Outcomes were measured at baseline and immediately following the 10–12-week intervention. Patient-reported outcomes included participant characteristics, overall well-being, cognition, fatigue, symptom severity, exercise barrier self-efficacy, and program satisfaction. Physical function included shoulder flexion, 30-s sit to stand, sit and reach, 2-min step test or 6-min walk test (in-person only), and single leg balance. Wilcoxon signed-rank tests were used to assess changes in patient-reported outcomes and physical function assessments from baseline to 12-weeks.

**Results:**

A total of 804 participants enrolled in the study in the first 2.5-years, with 699 completing the intervention. Wilcoxon signed rank tests and Rosenthal coefficients (*r*) showed significant (*p* ​< ​0.05) small improvements in well-being (*r* ​= ​0.10), fatigue (*r* ​= ​0.25), symptom severity (*r* ​= ​0.17), and self-efficacy (*r* ​= ​0.11). Significant (*p* ​< ​0.01) moderate to large improvements were observed for the 30-s sit to stand *(r* ​= ​0.54), sit and reach (left: *r* ​= ​0.46; right: *r* ​= ​0.41), 2-min step test (*r* ​= ​0.66), 6-min walk test (*r* ​= ​0.52), and single leg balance (left: *r* ​= ​0.32; right: *r* ​= ​0.34) assessments. Participants reported high satisfaction with program staff (average ​= ​4.5/5) and that the program was beneficial and enjoyable (average ​= ​4.6/5).

**Conclusion:**

EXCEL's group-based exercise program with behaviour change support, delivered in an online supervised setting to individuals living with cancer, may improve patient-reported outcomes and physical function and is associated with high participant satisfaction.

## 1 Introduction

Exercise is an evidenced based intervention for those living with and beyond cancer. Currently, there are exercise guidelines from leading international organizations, such as the American College of Sports Medicine [1] and American Cancer Society [2], as well as recommendations published in 2022 by the American Society of Clinical Oncology [3]. Despite these guidelines and recommendations, physical activity levels and maintenance of physical activity behaviour for individuals diagnosed with cancer remain low [4,5]. This is critical to address given the physical and psychosocial benefits of exercise and physical activity, such as improved physical fitness and function, fatigue, quality of life, depression, and anxiety [1]. One factor that may contribute to reduced physical activity but has received relatively little attention is geographic location. For example, those diagnosed with cancer from rural/remote communities experience geographic isolation and increased travel distances to medical facilities, which in turn may contribute to reduced access to cancer care services that would provide support for healthy lifestyle behaviours [[6], [7], [8]]. These barriers have been suggested to contribute to the higher prevalence rates of cancer diagnoses in rural and remote areas across Canada (944.5 per 100,000 people diagnosed with cancer in rural/remote areas vs 805.9 per 100,000 in urban areas) [9].

There is clearly a need for innovative exercise interventions that can reach rural/remote communities while also providing behaviour change education to support physical activity behaviour change and maintenance. Exercise oncology interventions delivered via online platforms (i.e., Zoom, Telehealth) may address this gap provided that they can reach individuals that otherwise may not be able to access in-person exercise sessions. Recent evidence has shown online exercise oncology interventions are not only safe and feasible, but also provide physical and psychosocial benefits [[10], [11], [12], [13]]. However, these studies were comprised of small sample sizes (n ​= ​15–44 participants) from urban areas; thus, there is a need to evaluate online exercise interventions in larger samples that include individuals with cancer from rural and remote communities.

The EXercise for Cancer to Enhance Living Well (EXCEL) study [14] is a hybrid effectiveness-implementation de-centralized clinical trial designed to better connect those living with cancer from rural and remote communities with exercise oncology programming and health behaviour change support. EXCEL is currently in its third year of recruitment and will continue to recruit into 2025. We have recently published results on EXCEL's first year of implementation, guided by the RE-AIM framework [15], focusing on the study's reach, adoption, and overall implementation [16]. The purpose of this interim analysis is to evaluate EXCEL's secondary effectiveness outcomes of patient-reported outcomes, physical function, and patient-reported satisfaction for the first 50% of the target sample (∼750 enrolled participants out of 1500 across 2.5-years). The primary outcome of self-reported and objectively measured physical activity will be examined only in the final analyses with the complete sample. This interim analysis fills an important gap in the current exercise oncology literature by addressing questions surrounding the effectiveness of online exercise on patient-reported and physical function outcomes that ultimately contribute to the health-related quality of individuals living with and beyond cancer.

## 2 Methods

### 2.1 Study design

**Fig. 1:**
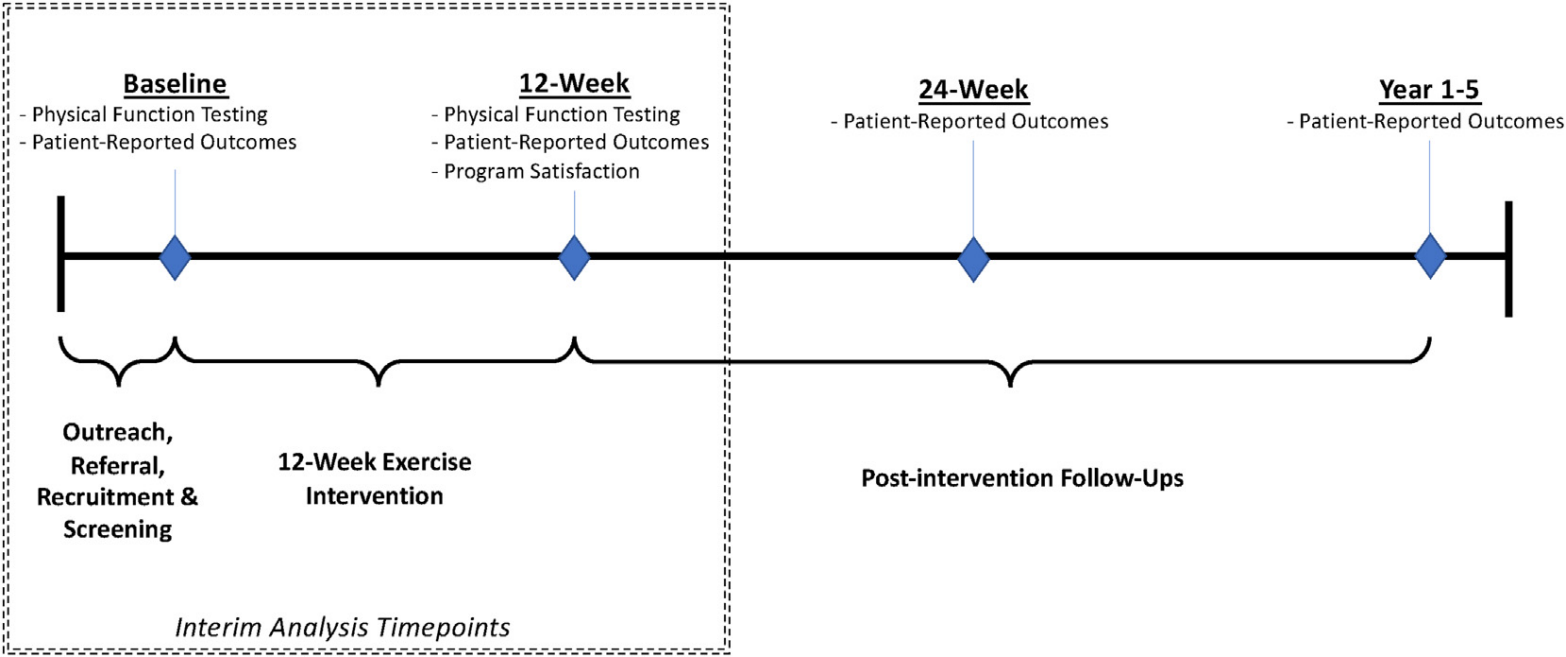
EXCEL study assessment timeline.

EXCEL is a 5-year hybrid effectiveness-implementation study in Canada that began in the Fall of 2020 and will continue recruitment through 2025. EXCEL offers a 10-12-week exercise intervention delivered primarily online, but also in-person in some rural areas [14]. Both online and in-person classes were included in the original study design. This analysis includes primarily those who participated in online classes due to public health restrictions related to COVID-19. Assessments are also conducted primarily online, and in-person for those sites that are able to accommodate. Briefly, study assessments (i.e., patient-reported outcomes, physical function testing) occur at baseline and immediately post-intervention (10-12-week timepoint); and for patient-reported outcomes only at 24-weeks post-intervention and annually for up to five years. Patient-reported satisfaction surveys occur at the 12-week timepoint. This interim analysis includes secondary outcome data from the baseline and 12-week timepoints, as well as satisfaction data at 12-weeks, for participants enrolled in Fall 2020 through Winter 2023 (Fig. 1). Ethics approval was granted from the Cancer Control Health Research Ethics Board of Alberta (HREBA.CC-20-0098), and each hub site across Canada received an associated ethics approval from their governing ethics board.

### 2.2 Participants

Participants were eligible to enroll in the EXCEL study if they met the following criteria: (1) ≥18 years with any cancer diagnosis; (2) pre-, currently receiving, or post-treatment up to 3 years^1^; (3) able to participate in mild exercise and physical activity two times per week; (4) currently residing in a rural community (population <100,000 people) or an urban community where exercise oncology resources were limited due to COVID-19 restrictions (2020–21 only); and (5) access to reliable internet to participate in live online exercise classes. Participant recruitment and screening for exercise participation was conducted online by study team Clinical Exercise Physiologists (CEPs) at the hub sites of Calgary, Halifax, and Toronto. Interim analyses include participants recruited from these respective hubs residing in British Columbia, Alberta, Saskatchewan, Manitoba, Ontario, Quebec, New Brunswick, Nova Scotia, Prince Edward Island, Newfoundland and Labrador, Yukon, and Northwest Territories (See Table 1). Screening included a review of the participant's medical history (cancer-related, treatment-related, chronic conditions), and an assessment of their physical activity readiness via the Physical Activity Readiness Questionnaire+ (PARQ+) [17]. Participants provided informed consent prior to participating in any study-related activities.

**Table 1:** Participant demographics and clinical characteristics (N = 699).

Variable	Number (%)
**Age (years)**^a^	57.9 (11.7)
**Location**	
Rural/Remote	498 (71.2)
Urban	201 (28.8)
**Exercise Class Delivery**	
Online	598 (85.6)
In-Person	101 (14.4)
**Province**	
British Columbia	30 (4.3)
Alberta	106 (15.2)
Saskatchewan	95 (13.6)
Manitoba	21 (3.0)
Ontario	227 (32.5)
Quebec	3 (0.4)
New Brunswick	17 (2.4)
Nova Scotia	185 (26.5)
Prince Edward Island	1 (0.1)
Newfoundland & Labrador	6 (0.9)
Yukon	1 (0.1)
Northwest Territories	7 (1.0)
**Sex**	
Female	585 (83.7)
Male	109 (15.6)
No information provided	5 (0.7)
**Ancestral Ethnicity**	
Aboriginal	10 (1.4)
African	3 (0.4)
Arab	2 (0.3)
Asian	34 (4.9)
British	194 (27.8)
Caribbean	3 (0.4)
European	190 (27.2)
Latin/Central and South America	7 (1.0)
Multi-ethnic	129 (18.5)
No information provided	53 (7.6)
Other	74 (10.6)
**Education**	
Some High School	11 (1.6)
Completed High School	54 (7.7)
Some University/College	109 (15.6)
Completed University/College	362 (51.8)
Some Graduate School	21 (3.0)
Completed Graduate School	137 (19.6)
No information provided	5 (0.7)
**Employment Status**	
Full-Time	167 (23.9)
Part-Time	260 (37.2)
Retired	73 (10.4)
Homemaker	23 (3.3)
Disability	141 (20.2)
Temporarily Unemployed	30 (4.3)
No information provided	5 (0.7)
**Marital Status**	
Never Married	60 (8.6)
Married	453 (64.8)
Common Law	71 (10.2)
Separated	23 (3.3)
Widowed	33 (4.7)
Divorced	54 (7.7)
No information provided	5 (0.7)
**Income**	
< $20,000	29 (4.1)
$20,000 – $39,999	66 (9.4)
$40,000 - $59,999	89 (12.7)
$60,000 – $79,999	105 (15.0)
$80,000 - $99,999	111 (15.9)
> $100,000	231 (33.0)
No information provided	68 (9.7)
**Cancer Type**	
Breast	347 (49.6)
Lung	56 (8.0)
Hematologic	56 (8.0)
Gynecological	47 (6.7)
Prostate	43 (6.2)
Skin	21 (3.0)
Brain	11 (1.6)
Colon	20 (2.9)
Head and Neck	7 (1.0)
Multiple Diagnoses	6 (0.9)
Other^b^	65 (9.3)
No information provided	20 (2.9)
**Advanced Cancer**	
Yes	156 (22.3)
No	538 (77.0)
No information provided	5 (0.7)
**Treatment Status**	
On	383 (54.8)
Off	311 (44.5)
No information provided	5 (0.7)
**Treatment Type for participants ‘On’ treatment**	
Chemotherapy	104 (27.2)
Radiation	12 (3.1)
Hormone Therapy	120 (31.3)
Biological Therapy	8 (2.1)
Combination^c^	61 (15.9)
Other^d^	75 (19.6)
No Information Provided	3 (0.8)

a *Age is expressed as mean (standard deviation)*.

b *Other = bladder, appendiceal, kidney, pancreatic, small bowel, colorectal, gastric, rectal, esophagus, or multiple cancer diagnoses*.

c Combination = any combination of chemotherapy, radiation, hormone therapy, or “other”.

d Other = immunotherapy, targeted therapy, surgery, antimetabolites.

### 2.3 Exercise intervention

Delivered by Qualified Exercise Professionals (QEPs) [14], exercise classes (∼8–15 participants) occurred twice per week for 60-min per session. All classes followed the same progression, which included a full-body warm-up (5–10 ​min), circuit exercise training with aerobic, resistance, and balance exercises (45–50 ​min), and a cool-down consisting of full-body stretching (5–10 ​min). The majority of group exercise classes took place online via Zoom. In limited locations in the Maritimes, in-person classes occurred in rural areas where the catchment was sufficient to deliver classes with a minimum of 8 participants. Adherence to the exercise intervention was assessed by tracking the total number of classes attended in comparison to the total number of possible classes. If physician clearance for exercise participation was deemed necessary by the CEP during screening or if interest in the EXCEL study was expressed late, participants were allowed to join EXCEL's exercise intervention up to 2-weeks after the start. Thus, the total possible exercise classes ranged from 20 to 24 classes (i.e., 10–12 weeks of exercise delivery) and total adherence was reported as a percentage of exercise classes attended.

To deliver additional exercise oncology behaviour change support, EXCEL utilizes an “Exercise and Educate” model that focuses on the training of QEPs to facilitate the following educational topics: (1) Principles of Exercise; (2) Self-Monitoring for Physical Activity; (3) Physical Activity Goal Setting; (4) Behaviour Change and Relapse Prevention; (5) Fatigue and Stress Management; and (6) Social Support and Long-Term Physical Activity. Exercise and Educate includes educational handouts, QEP-facilitated discussion of each education topic during and after designated exercise classes, and live exercise educational webinars for participants (optional attendance) delivered by study staff every two weeks throughout the 12-week intervention. Participants were also given access to the recorded webinars following the live presentation. Additional details regarding EXCEL's 12-week Exercise and Educate intervention can be referred to in the previously published protocol manuscript [14].

### 2.4 Measures

#### 2.4.1 Patient-reported outcomes

Patient-reported outcomes were completed online via Research Electronic Data Capture (REDCap) [18,19]. Demographic and clinical characteristics were self-reported and included age, location (i.e., rural vs urban), intervention delivery mode (i.e., online vs in-person), biological sex, ancestral ethnicity, education status, employment status, and marital status. Clinical characteristics included cancer type, treatment status, and treatment type. For this interim analysis, the following secondary outcome measures were analyzed, using reliable and validated patient-reported outcome measurements: Functional Assessment of Cancer Therapy – General (FACT-G) [20], Functional Assessment of Cancer Therapy – Cognition (FACT-Cog) [21], Functional Assessment of Chronic Illness Therapy – Fatigue (FACIT-F) [22], Edmonton Symptom Assessment Scale - Revised (ESAS-r) [23], and the Exercise Barrier Self-Efficacy questionnaire [24].

##### 2.4.1.1 FACT-G

The FACT-G is a 27-item questionnaire that assesses well-being through four sub-domains: physical, social/family, emotional, and functional well-being. Each item was answered on a five-point Likert scale (i.e., 0 ​= ​not at all and 4 ​= ​very much), and the recall period was seven days. A final score was calculated from the sum of each sub-domain score (range 0–108) and was representative of overall well-being. Higher scores represent better overall well-being.

##### 2.4.1.2 FACT-Cog

The FACT-Cog is a 37-item questionnaire that assesses cognition through four sub-domains: perceived cognitive impairment, perceived comments from others, perceived cognitive ability, and impact on quality of life. Each item was answered on a five-point Likert scale (i.e., 0 ​= ​not at all and 4 ​= ​very much or 0 ​= ​several times a day and 4 ​= ​never), and the recall period was seven days. A final score was calculated from the sum of each sub-domain score (range 0–148) and was representative of overall cognitive function. Higher scores represent better overall cognitive function.

##### 2.4.1.3 FACIT-F

The FACIT-F consists of 13-items to assess fatigue severity in the last seven days. Each of the 13 items was answered on a five-point Likert scale (i.e., 0 ​= ​not at all and 4 ​= ​very much). All items are summed to generate a total score on a scale of 0–52. Higher scores represent less fatigue severity.

##### 2.4.1.4 ESAS-r

The ESAS-r assesses symptom burden from nine cancer related symptoms. Symptoms include pain, tiredness, drowsiness, nausea, appetite, shortness of breath, feeling sad, feeling nervous, and well-being. Each symptom was rated on a scale of 0–10 (0 ​= ​no burden and 10 ​= ​extreme burden). All symptom ratings were summed together resulting in a total symptom burden score ranging from 0 to 90. Higher scores represent greater symptom burden.

##### 2.4.1.5 Exercise Barrier Self-Efficacy

The Exercise Barrier Self-Efficacy questionnaire asks participants to rate their self-efficacy for participating in exercise on their own. Participants rank their exercise barrier self-efficacy across 9-items, which include responding to the prompt “Exercise when…”: “you lack discipline”, “nauseated”, “it's not a priority”, “the weather is bad”, “you are tired”, “you are not interested”, “you lack time”, “you don't enjoy it”, and “no one is there to encourage you”. All items were rated from 0 to 100% at 10% intervals. Items were summed together and averaged to generate a total exercise barrier self-efficacy score. Interpretation of the scale was as follows: 0–20% ​= ​not at all confident; 20–40% ​= ​slightly confident; 40–60% ​= ​moderately confident; 60–80% ​= ​very confident; and 80–100% ​= ​extremely confident. Higher scores represent greater exercise barrier self-efficacy.

#### 2.4.2 Physical function

Specific details regarding each physical function assessment can be found in EXCEL's previously published protocol manuscript [14]. Briefly, the following data were collected: (1) self-reported height (centimeters) and weight (kilograms) for the calculation of body mass index; (2) shoulder flexion range of motion reported in degrees (range ​= ​0–180°) [25]; (3) 30-s sit to stand reported as the total number of full sit to stands within a 30-s timeframe [26,27]; 4) sit and reach assessment reported in centimeters [28]; 5) 2-min step test reported as the total number of steps taken in a 2-min timefame [29] or 6-min walk test (in-person only) reported as total distance walked in 6-min; and 6) single-leg balance reported as the total time, in seconds, able to balance on one leg [30]. All assessments were conducted online via Zoom and included one lead assessor (CEP) and one secondary assessor (trained QEP) to ensure participant safety through additional monitoring and to confirm collected data with the primary assessor. If a participant was unable to complete a physical function assessment due to physical limitations, the respective assessment was skipped for that participant.

#### 2.4.3 Patient-reported satisfaction

An online satisfaction survey was distributed to each participant at the conclusion of the 12-week intervention via REDCap [18,19]. The satisfaction survey consists of 33-items, each of which were rated on a five-point Likert scale (i.e., 1 ​= ​not at all and 5 ​= ​very much). Topics included (1) participant experience regarding EXCEL program and staff; (2) how participants felt about the EXCEL exercise program; (3) participant plans for continuing exercise in the year after the EXCEL exercise program; (4) EXCEL program/study burden on participants; and (5) participant reflection on program participation. Each item was scored individually and reported descriptively.

### 2.5 Statistical analysis

Statistical analyses were performed with RStudio 1.3 (Boston, MA, USA). Descriptive statistics expressed as mean ​± ​standard deviation (SD) or number (%) were used to describe sample characteristics as well as patient-reported experience survey responses. Independent t-tests and chi-square tests of proportions were used to compare demographics, clinical characteristics, and baseline outcomes between rural and urban participants as well as between online and in-person participants. A complete case analysis with listwise deletion was used for handling missing data prior to conducting any analyses. Shapiro–Wilk tests were used to assess data normality. Wilcoxon signed-rank tests were used to assess changes in patient-reported outcomes and physical function assessments from baseline to 12-weeks. The Rosenthal coefficient (r) was used to calculate effect sizes for within group differences and was interpreted with the following categories: small effect, <0.30; moderate effect, 0.30 ​< ​0.50; large effect, >0.50. The alpha level was set a priori for all statistical analyses at *p* ​< ​0.05.

## 3 Results

### 3.1 Study enrollment, demographics, and clinical characteristics

**Fig. 2:**
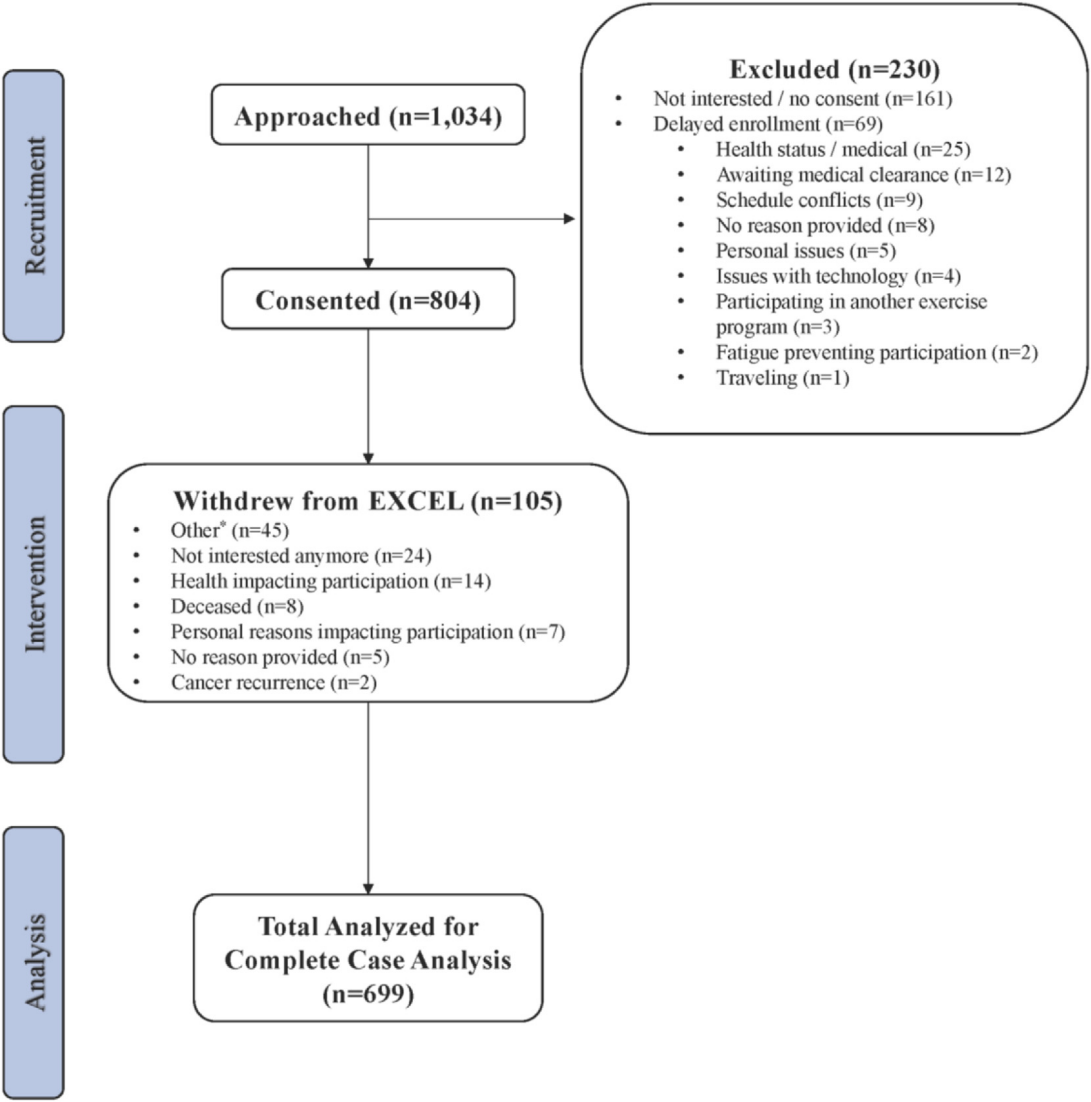
CONSORT (Consolidated Standards of Reporting Trials) diagram. Other∗ = time commitment symptom burden, family obligations, physical limitations

A total of 804 individuals living with and beyond cancer agreed to participate in the EXCEL study from Fall 2020 until Winter 2023 (8 total 12-week intervention waves delivered), with 699 participants completing EXCEL's 12-week exercise program. Participants were from 12 Canadian provinces, recruited via the 3 hub sites of Calgary, Halifax, and Toronto. Refer to Fig. 2 (CONSORT Diagram) for study recruitment, enrollment, and dropout numbers/reasons for withdrawing. In this interim analysis, 71.2% were from rural areas while 28.8% were from urban areas that participated only during the COVID-19 2020–21 restrictions, in which urban participants had no other access to exercise oncology resources. An overall adherence rate of 80.1% was reported for the 12-week exercise program.

Demographics and clinical characteristics (Table 1) indicate EXCEL participants were predominantly female (83.7%), had a university/college degree or higher (74.4%), were employed full- or part-time (61.1%), and primarily participated in EXCEL's online exercise classes (85.6%). Clinically, the majority of participants reported an early-stage cancer (77.0%) and received a breast cancer diagnosis (49.6%). Most participants were also actively receiving treatment (54.8%), which primarily included chemotherapy (27.2%), hormone therapy (31.3%), or a combination of different treatments (15.9%). Age, sex, education, income, and employment status significantly differed (p ​< ​0.05) between rural and urban participants. At baseline, overall well-being (FACT-G total score), sit and reach on the left leg, and single leg balance on both legs significantly differed between rural and urban participants. Income and single leg balance on both legs were the only variable that significantly differed (p ​< ​0.05) between online and in-person participants. Refer to Supplemental Table 1 for group (rural vs urban; online vs in-person) differences.

### 3.2 Patient-reported outcomes

Wilcoxon signed ranked tests revealed statistically significant improvements in the distribution of total scores (Table 2 and Supplemental Fig. 1) for the FACT-G (median score: Baseline ​= ​78.0, 12-week ​= ​80.0, *r* ​= ​0.10, *p* ​< ​0.01), FACIT-Fatigue (median score: Baseline ​= ​39.0, 12-week ​= ​41.0, r ​= ​0.25, p ​< ​0.01), and the ESAS-r (median score: Baseline ​= ​15.0, 12-week ​= ​14.0, *r* ​= ​0.17, *p* ​< ​0.01). Statistically significant improvements in the distribution of scores for total exercise barrier self-efficacy were also observed (median score: Baseline ​= ​47.8%, 12-week ​= ​51.1%, *r* ​= ​0.11 *p* ​= ​0.01). Cognition did not significantly improve (median score: Baseline ​= ​114.0, 12-week ​= ​112.5, *r* ​= ​0.01, *p* ​= ​0.72).

**Table 2:** EXCEL Baseline and 12-Week Outcome Results – median (interquartile range).

Variable	N	Baseline	12-Weeks	*p-value*	Effect Size (r)
**Patient-Reported Outcomes**					
**FACT-General (0–108)**^a^	574	78.0 (20.0)	80.0 (19.0)	0.01∗	0.10
Physical Well-Being (0–28)^a^	577	23.0 (6.0)	24.0 (6.0)	0.05	0.08
Social/Family Well-Being (0–24)^a^	576	20.0 (7.0)	19.0 (7.0)	0.03∗	0.09
Emotional Well-Being (0–28)^a^	578	19.0 (6.0)	19.0 (5.0)	<0.01∗	0.12
Functional Well-Being (0–28)^a^	579	18.0 (8.0)	19.0 (7.0)	<0.01∗	0.18
**FACT-Cognition (0–148)**^a^	570	114.0 (37.8)	112.5 (38.0)	0.72	0.01
Perceived Cognitive Impairment (0–72)^a^	575	63.0 (23.0)	61.0 (23.0)	0.10	0.07
Impact on Quality of Life (0–16)^a^	574	12.0 (7.0)	13.0 (7.0)	<0.01∗	0.22
Comments from Others (0–16)^a^	577	16.0 (1.0)	16.0 (1.0)	0.60	0.04
Perceived Cognitive Abilities (0–28)^a^	574	19.0 (10.0)	19.0 (9.0)	0.68	0.02
**FACIT-Fatigue (0–52)**^a^	569	39.0 (15.0)	41.0 (13.0)	<0.01∗	0.25
**Edmonton Symptom Assessment Scale-Revised (0–90)**^b^	574	15.0 (17.0)	14.0 (15.0)	<0.01∗	0.17
Pain (0–10)^b^	574	1.0 (3.0)	2.0 (3.0)	0.20	0.03
Tiredness (0–10)^b^	574	3.0 (4.0)	2.0 (3.0)	<0.01∗	0.29
Drowsiness (0–10)^b^	574	2.0 (4.0)	1.0 (3.0)	<0.01∗	0.15
Nausea (0–10)^b^	574	0.0 (0.0)	0.0 (0.0)	0.18	0.07
Appetite (0–10)^b^	574	0.0 (1.0)	0.0 (0.8)	<0.01∗	0.14
Shortness of breath (0–10)^b^	574	0.0 (1.0)	0.0 (1.0)	0.22	0.04
Feeling Sad (0–10)^b^	574	1.0 (3.0)	1.0 (3.0)	0.15	0.06
Feeling Nervous (0–10)^b^	574	1.0 (3.0)	1.0 (3.0)	0.26	0.05
Well-Being (0–10)^b^	574	3.0 (4.0)	2.0 (4.0)	<0.01∗	0.13
**Exercise Barrier Self-Efficacy (0–100%)**^a^	577	47.8 (28.9)	51.1 (26.7)	0.01∗	0.11
**Physical Function Measures**					
**30-Second Sit to Stand**					
Total # of sit-to-stands^a^	572	13.0 (5.0)	14.0 (5.0)	<0.01∗	0.54
**2-Minute Step Test**					
Total # of steps taken on right leg^a^	517	72.0 (29.0)	86.0 (25.0)	<0.01∗	0.66
**6-Minute Walk Test**					
Total distance walked in 6-min (meters)^a^	42	501.8 (146.5)	557.0 (218.5)	<0.01∗	0.52
**Single Leg Balance**					
Left Leg (sec)^a^	575	36.2 (29.9)	45.0 (19.8)	<0.01∗	0.32
Right Leg (sec)^a^	574	40.0 (28.3)	45.0 (18.2)	<0.01∗	0.34
**Shoulder Flexion Range of Motion**					
Left Arm (0–180°)^a^	566	163.0 (21.4)	165.0 (19.0)	<0.01∗	0.18
Right Arm (0–180°)^a^	566	164.0 (20.5)	165.5 (19.9)	<0.01∗	0.16
**Lower Body Flexibility**					
Left Leg (cm)^a^	552	0.0 (16.5)	2.7 (15.0)	<0.01∗	0.46
Right Leg (cm)^a^	553	0.0 (16.5)	2.5 (14.5)	<0.01∗	0.41

∗*p < 0.05* from Wilcoxon Signed Rank Test.

*Abbreviation Notes:* ‘SD’ = standard deviation; ‘°’ = degrees; ‘cm’ = centimeter; ‘sec’ = seconds; ‘r’ = Rosenthal Coefficient.

a Higher score = better outcome.

b Higher score = worse outcome.

### 3.3 Physical function

Wilcoxon signed ranked tests revealed statistically significant improvements (p ​< ​0.01 for all measures) in the distribution of total scores (Table 2 and Supplemental Figs. 2–5) for 30-s sit to stand (median score: Baseline ​= ​13.0 sit to stands, 12-week ​= ​14.0 sit to stands, *r* ​= ​0.54), 2-min step test (median score: Baseline ​= ​72.0 steps, 12-week ​= ​86.0 steps, *r* ​= ​0.66), 6-min walk test (median score: Baseline ​= ​501.8 ​m, 12-week ​= ​557.0 ​m, *r* ​= ​0.52), single leg balance (median score left leg: Baseline ​= ​36.2 ​s, 12-week ​= ​45.0 ​s, *r* ​= ​0.32; median score right leg: Baseline ​= ​40.0 ​s, 12-week ​= ​45.0 ​s, *r* ​= ​0.34), shoulder flexion range of motion (median score left arm: Baseline ​= ​163.0°, 12-week ​= ​165.0°, *r* ​= ​0.18; median score right arm: Baseline ​= ​164.0°, 12-week ​= ​165.5°, *r* ​= ​0.16), and lower body flexibility (median score left leg: Baseline ​= ​0.0 ​cm, 12-week ​= ​2.7 ​cm, *r* ​= ​0.46; median score right leg: Baseline ​= ​0.0 ​cm, 12-week ​= ​2.5 ​cm, *r* ​= ​0.41).

### 3.4 Patient-reported satisfaction

In total, 570 participants provided responses to the online satisfaction survey, with higher scores reflecting a more positive experience/higher satisfaction (Table 3). For experience with the EXCEL staff, participants on average indicated “quite a bit” or “very much” (average score ​= ​4.6/5) when asked if program staff were knowledgeable, supportive, assisted them with meeting their health and wellness goals, and cared about personal health and well-being. For experience with the EXCEL exercise program, participants on average indicated “quite a bit” or “very much” (average score ​= ​4.6/5) when asked if they felt the exercise program was beneficial, enjoyable, and motivating. Participants also found the exercise program to be rewarding and useful, both in terms of personal benefit from the exercise program as well as the study results helping others (average score ​= ​4.5/5).

**Table 3:** Patient Reported Satisfaction – mean (standard deviation).

Satisfaction Survey Question	Average Survey Response^c^ (N = 570)
**Experience regarding EXCEL staff**	
*Completing the EXCEL exercise program helped me to meet my health and wellness goals.*^a^	4.3 (0.8)
*The program helped me manage symptoms and side effects related to my cancer/treatment.*^a^	3.7 (1.1)
*The program helped increase my knowledge related to benefits of physical activity for cancer survivors.*^a^	4.2 (0.9)
*The program staff made me feel comfortable.*^a^	4.9 (0.4)
*The program staff were knowledgeable.*^a^	4.9 (0.4)
*The program staff were informative and made an effort to teach me about my health and wellness.*^a^	4.8 (0.5)
*The program staff helped me to meet my health and wellness goals.*^a^	4.5 (0.7)
*The program staffed seemed to care about my personal health and wellbeing.*^a^	4.8 (0.5)
*The program staff were supportive.*^a^	4.9 (0.4)
*The program staff worked with me to ensure the exercises were appropriate.*^a^	4.8 (0.6)
**How participants felt about the EXCEL exercise program**	
*How beneficial was the exercise program?*^a^	4.7 (0.6)
*How enjoyable was the exercise program?*^a^	4.6 (0.6)
*How supportive were your family/friends of the exercise program?*^a^	4.5 (0.8)
*How motivated were you to do the exercise program?*^a^	4.4 (0.7)
*How difficult was it for you to do the exercise program?*^b^	2.4 (1.0)
**Participant plans for continuing exercise in the year after the EXCEL exercise program**	
*How beneficial do you think it will be for you to continue exercising?*^a^	4.7 (0.5)
*How enjoyable do you think it will be for you to continue exercising?*^a^	4.2 (0.8)
*How supportive do you think your family/friends will be if you continue exercising?*^a^	4.5 (0.8)
*How motivated are you to continue to exercise?*^a^	4.3 (0.8)
*How difficult do you think it will be for you to continue exercising on your own?*^b^	2.5 (1.0)
*How confident are you to continue exercising on your own?*^a^	3.7 (1.0)
*Do you feel that the EXCEL program prepared you to exercise on your own?*^a^	4.2 (0.9)
**Participant burden – “How much of a burden was it for you to complete each of the following:”**	
*The aerobic fitness test.*^b^	1.7 (0.9)
*The muscular strength, balance, and flexibility test.*^b^	1.7 (1.0)
*The online questionnaires.*^b^	1.6 (0.9)
*The supervised exercises sessions.*^b^	1.4 (0.8)
*Traveling to and from the exercise facility.*^b^	1.1 (0.5)
**Participant reflection on participation.**	
*It was rewarding.*^a^	4.7 (0.6)
*It was a waste of time.*^b^	1.0 (0.3)
*It will be useful for research helping others.*^a^	4.4 (0.8)
*It was useful for me personally.*^a^	4.7 (0.6)
*I would recommend the exercise program to other cancer patients.*^a^	4.9 (0.4)

*Abbreviation Notes:* ‘SD’ = Standard Deviation.

a Higher score = positive response.

b Higher score = negative response.

c Survey Scale: 1 = ‘not at all’; 2 = ‘a little bit’; 3 = ‘somewhat’; 4 = ‘quite a bit’; 5 = ‘very much’.

## 4 Discussion

This interim analysis examined EXCEL's secondary effectiveness outcomes (i.e., patient-reported outcomes, physical function, and patient-reported satisfaction) for the first 50% of the estimated total sample size. A total of 699 individuals living with and beyond cancer from 12 Canadian provinces were included in the complete case analysis (from 804 recruited), with 498 participants from rural areas and 201 participants from urban areas. There were 598 participants online, and 101 participants that participated in EXCEL in-person exercise classes across the Maritime regions from rural communities only. There were no in-person urban participant programs. For the purposes of the interim analyses, no sub-group analyses or inclusion of delivery mode or differences in baseline outcomes as covariates were performed. These by group or covariate analyses will be included in the full sample size analyses following EXCEL recruitment.

In the total interim analysis sample, improvements were observed in participants’ well-being, fatigue, symptom severity, exercise barrier self-efficacy, and all assessments of physical function. Program satisfaction was also positive, with the majority of participants feeling the exercise was beneficial while simultaneously reporting that study participation was not burdensome. In line the with RE-AIM framework [15], this analysis highlights the potential physical and psychosocial benefits of the EXCEL hybrid effectiveness-implementation study. These results supplement first-year findings that showed successful reach, adoption, and implementation of the EXCEL study in rural and remote communities [16]. Continued monitoring of RE-AIM constructs, including effectiveness, provides quality improvement feedback to the study team, which informs implementation strategies throughout the 5-year study with the goal ultimately of delivering an effective and sustainable community-based exercise oncology program.

Our study found that the greatest improvements in patient-reported outcomes were observed in fatigue/tiredness, reporting effect sizes of 0.25 and 0.29, respectively. Though these changes are considered ‘small’ effects, it's important to note that these results are in line with previous work exploring the effects of community-based exercise on fatigue in cancer populations. In a recent meta-analysis, Wagoner and colleagues [31] reported a significant improvement in fatigue from 12 community-based exercise oncology interventions (pooled effect size of 0.30). Furthermore, previous work from Mustian et al., in 2017 [32] studying the same effect in randomized controlled trials reported similar results for the impact of exercise on fatigue (effect size of 0.30). Our findings further support the use of exercise in primarily online settings to alleviate cancer-related fatigue.

Improvements in overall well-being, symptom severity, and exercise barrier self-efficacy are consistent with previous studies of in-person community-based exercise oncology interventions [[33], [34], [35], [36]]; however these findings vary when compared to previous online exercise oncology interventions. For example, previous studies show no improvement in symptom severity as measured by the ESAS-r [11]. While this study's intervention did include behaviour change support, there was low attendance to the optional educational sessions (15–49%) which took place at a separate time to the online exercise class. In contrast, EXCEL's “Exercise and Educate” intervention approach includes delivering the program within a positive motivational climate, with groups of at least 8-participants exercising together and discussing cancer-related experiences with additional behaviour change education during the exercise classes. These intervention characteristics are similar to those of other work reporting benefits of online exercise oncology programs in which behaviour change education and self-regulatory skills are introduced and discussed as a group during an exercise session [12]. As a cost-effective way to support behaviour change, further work on training QEPs to embed behaviour change skill instruction and using a health coaching framework to build positive communication should continue to be examined.

Physical function improved across all measures after the EXCEL exercise intervention. In particular, the 30-s sit to stand and the 2-min step test displayed ‘large’ effects (effect size ​= ​0.54 and 0.66, respectively). Specific to the 30-s sit to stand test, our finding is in line with previous work, where both Romero et al. [10] and Myers et al. [12] reported large effects (effect size ​= ​0.76 and 1.22 respectively) in chair sit to stands after 6-16-weeks of online exercise training in women with breast cancer. Similarly, Gell et al. [13] reported significant improvements in the 30-s sit to stand test in a sample of rural cancer survivors when compared to a waitlist control group after a supervised, online, group-based 16-week exercise intervention. The remaining physical function measures (i.e., shoulder range of motion, lower body flexibility, 6-min walk test, and single balance) all displayed significant improvements that ranged from small to moderate effects (effect size ​= ​0.16–0.52). In particular, participants in the present study improved their median 6-min walk test distance by 56 ​m, which fell within the minimal clinically important difference (41.5 ​m–66.5 ​m) reported within cancer populations [37]. Further, participants achieved a median distance of 557 ​m in the post-intervention assessment. This distance exceeds that of the average 6-min walk distances reported in a recent meta-analysis of breast cancer survivors (477 ​m) [38], as well as minimum distances that have been linked with mortality (300 ​m) [39] and increased hospitalization (468 ​m) [40] in individuals with cardiovascular disease. Taken together, these results support the literature that primarily online exercise training improves physical function [1,41,42].

Overall, satisfaction with the EXCEL study was positive, possibly contributing to the high adherence rate of 80.1% and reported benefits. Participants not only found the exercise program to be beneficial, enjoyable, and rewarding, but also helped prepare them for continuing to exercise after the program. Self-efficacy for continuing to exercise after EXCEL was moderate to high, as participants reported on average that they were ‘somewhat’ to ‘quite a bit confident’ in continuing to participate in exercise. Continued focus on supporting confidence to ‘see self as an exerciser’ will be reinforced in EXCEL. Behaviour change strategies such as providing choice within sessions, fostering success through progressive exercises, and highlighting changes in individual function over time, will be reinforced within the instructor training and delivery of EXCEL. Fostering a positive motivational climate within our ‘Exercise and Educate’ approach will be crucial to eliciting continued exercise behaviour change and longer-term maintenance.

As a whole, these findings remain encouraging as they speak to the effectiveness of the Exercise and Educate approach, including behaviour change support to help participants build the confidence and skills necessary to remain physically active long-term. While previous studies have also reported relatively high satisfaction with exercise oncology programs, there has been relatively lower reported satisfaction related to encouragement that is provided by instructors with remote/home-based interventions [43]. Importantly, this was not observed in the EXCEL study, as participants acknowledged that study staff were supportive, knowledgeable, and helped them to better understand their own health and wellness. This finding reflects the EXCEL training pathway, which provides extensive training and support to its QEPs [14], emphasizing both high quality exercise tailoring as well as how to deliver classes using the Exercise and Educate and the health coaching approach to support participant goals, exercise behaviour change, and overall well-being. These findings, though descriptive, may be important for future intervention development as they support the training of QEPs that then translates to effective exercise tailoring and behaviour change support.

Several limitations from our study should be considered. First, we conducted multiple hypothesis tests which leaves our results prone to type I error. However, the reported results are part of interim analyses, with only 50% of the expected overall sample and the goal of using these results as feedback to the program within the quality improvement cycles, thus informing continued implementation. Given our substantially larger sample size compared to similar previous work [[11], [12], [13]], we believe our analysis is sufficient to examine EXCEL secondary outcomes. Second, we used listwise deletion which may introduce bias into the statistical analyses. Yet, we also note that our missing data rate did not exceed 7% for any patient-reported or physical function outcome. Last, our sample included both rural and urban-based participants where differences in some baseline characteristics and outcomes were identified. Urban-based participants were allowed to join the EXCEL during COVID-19 lockdowns to provide safe access to exercise oncology resources. Though their inclusion may introduce the possibility of skewing the study's findings, our main objective of this analysis was to provide a broad preliminary overview on the effects of EXCEL's ongoing exercise intervention. Future analyses that include the complete dataset will conduct sensitivity analyses to account for the potential differences amongst rural and urban-based participants, as well as in-person vs online participation, and consider the identified variables as model covariates.

## 5 Conclusion

Findings from the EXCEL interim analysis showed positive effects across rural/remote (71.2%) and urban-based (28.8%) individuals living with and beyond cancer, as well as for both online (86%) and in-person (14%) participants. Notably, improvements were observed in overall well-being, fatigue, symptom severity, and physical function. These findings align with established research on community-based exercise interventions in cancer populations, supporting the effectiveness of group-based exercise programs that take place primarily online. High participant satisfaction and adherence to EXCEL's exercise intervention further underscore the program's effectiveness. The EXCEL study's emphasis on providing a positive motivational climate contributes to its effectiveness, which in turn highlights the potential for exercise interventions supplemented with behaviour change support to continue to improve the overall well-being and physical function of individuals living with and beyond cancer.

## Author contributions

Conceptualization: SNC-R and CW; Methodology: SNC-R, CW, and JD; Formal Analysis: CW; Data Curation: CW, JD, DS, JL; Writing – Original Draft Preparation: SNC-R and CW; Writing – Review & Editing: all authors.

## Funding

EXCEL is funded by a Canadian Institutes of Health Research and Canadian Institutes of Health Research and Canadian Cancer Society Survivorship Team Gran (Grant # 706673). Additional program funding provided by the 10.13039/501100000001Alberta Cancer Foundation (Grant #N/A).

## Institutional review board statement

The study was conducted according to the guidelines of the Declaration of Helsinki and approved by the Health Research Ethics Board of Alberta at the University of Calgary as the central institution in the multisite trial (HREBA.CC-20-0098; Approved June 9, 2020). All sites have respective provincial ethics approvals.

## Informed consent statement

Informed consent was obtained from all subjects involved in the study.

## Data availability statement

Data can be made available upon reasonable request.

## Declaration of competing interest

All authors declare no conflicts of interest.

## Footnotes

1 Eligibility can be beyond 3 years with a medical referral.
