# Supplementary material for: Exercise and behaviour change support for individuals living with and beyond cancer: Interim results and program satisfaction of the EXCEL study
Source: JSAMS Plus. 2024 Feb 24;3:100055. doi: 10.1016/j.jsampl.2024.100055 (PMC13008431; doi:10.1016/j.jsampl.2024.100055)

Supplemental Table 1. Rural vs Urban and Online vs In-Person Baseline Differences (p < 0.05)

| Variable | Rural vs Urban  mean (SD) or number (%) | |
| --- | --- | --- |
|  | Rural (n=498) | Urban (n=201) |
| Age (years)^†^ | 59.0 (12.7) | 54.2 (12.9) |
| Sex |  |  |
| Female | 401 (80.5) | 184 (91.5) |
| Male | 92 (18.5) | 17 (8.5) |
| No information provided | 5 (1.0) | 0 (0.0) |
| Education |  |  |
| Some High School | 9 (1.8) | 2 (1.0) |
| Completed High School | 48 (9.6) | 6 (3.0) |
| Some University / College | 84 (16.9) | 25 (12.4) |
| Completed University / College | 250 (50.2) | 112 (55.7) |
| Some Graduate School | 16 (3.2) | 5 (2.5) |
| Completed Graduate School | 86 (17.3) | 51 (25.4) |
| No information provided | 5 (1.0) | 0 (0.0) |
| Employment Status |  |  |
| Full Time | 110 (22.1) | 57 (28.4) |
| Part Time | 203 (40.8) | 57 (28.4) |
| Retired | 52 (10.4) | 21 (10.4) |
| Homemaker | 20 (4.0) | 3 (1.5) |
| Disability | 91 (18.3) | 50 (24.9) |
| Temporarily Unemployed | 17 (3.4) | 13 (6.5) |
| No information provided | 5 (1.0) | 0 (0.0) |
| Income |  |  |
| < $20,000 | 18 (3.6) | 11 (5.5) |
| $20,000 – $39,999 | 49 (9.8) | 17 (8.5) |
| $40,000 - $59,999 | 69 (13.9) | 20 (10.0 |
| $60,000 – $79,999 | 85 (17.1) | 20 (10.0) |
| $80,000 - $99,999 | 84 (16.9) | 27 (13.4) |
| > $100,000 | 151 (30.3) | 80 (39.8) |
| No information provided | 42 (8.4) | 26 (12.9) |
| FACT-General | 77.3 (14.8) | 74.7 (14.2) |
| Lower Body Flexibility |  |  |
| Left Leg (cm) | -0.8 (12.0) | -3.3 (14.3) |
| Single Leg Balance |  |  |
| Left Leg (sec) | 28.6 (16.4) | 33.6 (14.6) |
| Right Leg (sec) | 29.6 (16.0) | 33.4 (15.0) |
|  | **Online vs In-Person**  **mean (SD) or number (%)** | |
|  | Online (n=598) | In-Person (n=101) |
| Income |  |  |
| < $20,000 | 23 (3.8) | 6 (5.9) |
| $20,000 – $39,999 | 51 (8.5) | 15 (14.9) |
| $40,000 - $59,999 | 70 (11.7) | 19 (18.8) |
| $60,000 – $79,999 | 90 (15.1) | 15 (14.9) |
| $80,000 - $99,999 | 98 (16.4) | 13 (12.9) |
| > $100,000 | 210 (35.1) | 21 (20.8) |
| No information provided | 56 (9.4) | 12 (11.9) |
| Single Leg Balance |  |  |
| Left Leg (sec) | 30.9 (15.8) | 23.9 (17.0) |
| Right Leg (sec) | 31.4 (15.6) | 26.3 (16.2) |
| ^†^ *= Age is expressed as mean (standard deviation)* | | |

Supplemental Figure 1 A-F. Baseline and 12-Week Patient Reported Outcome Distributions


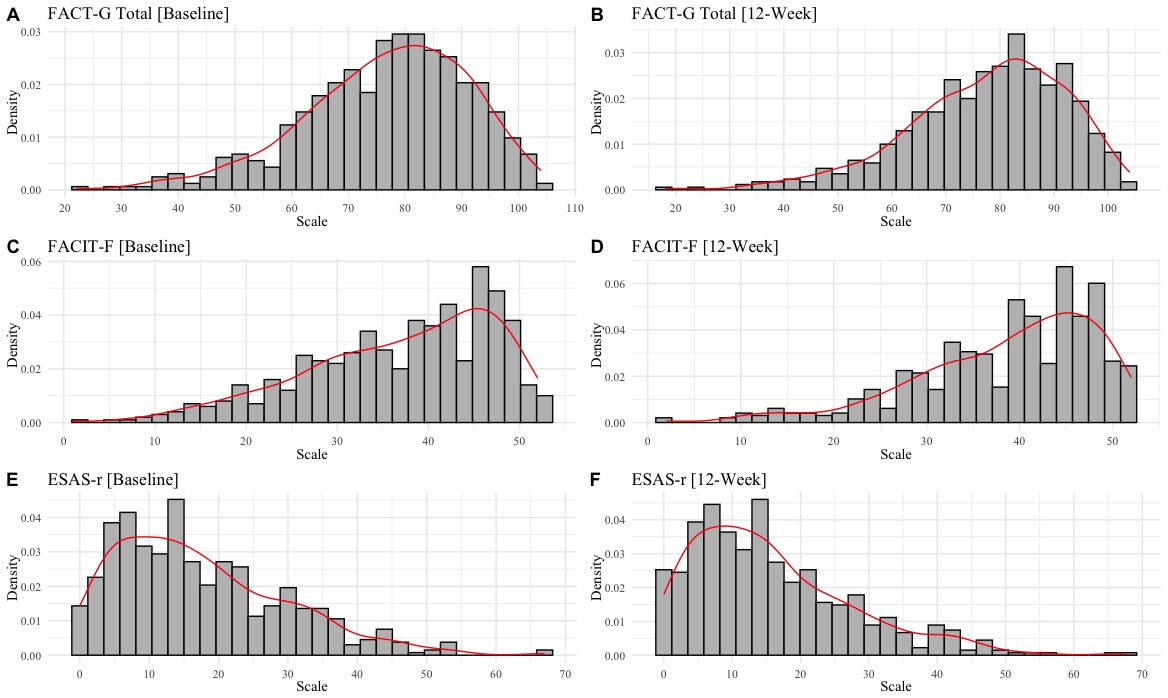


Supplemental Figure 2 A-D. Baseline and 12-Week Shoulder Flexion Range of Motion


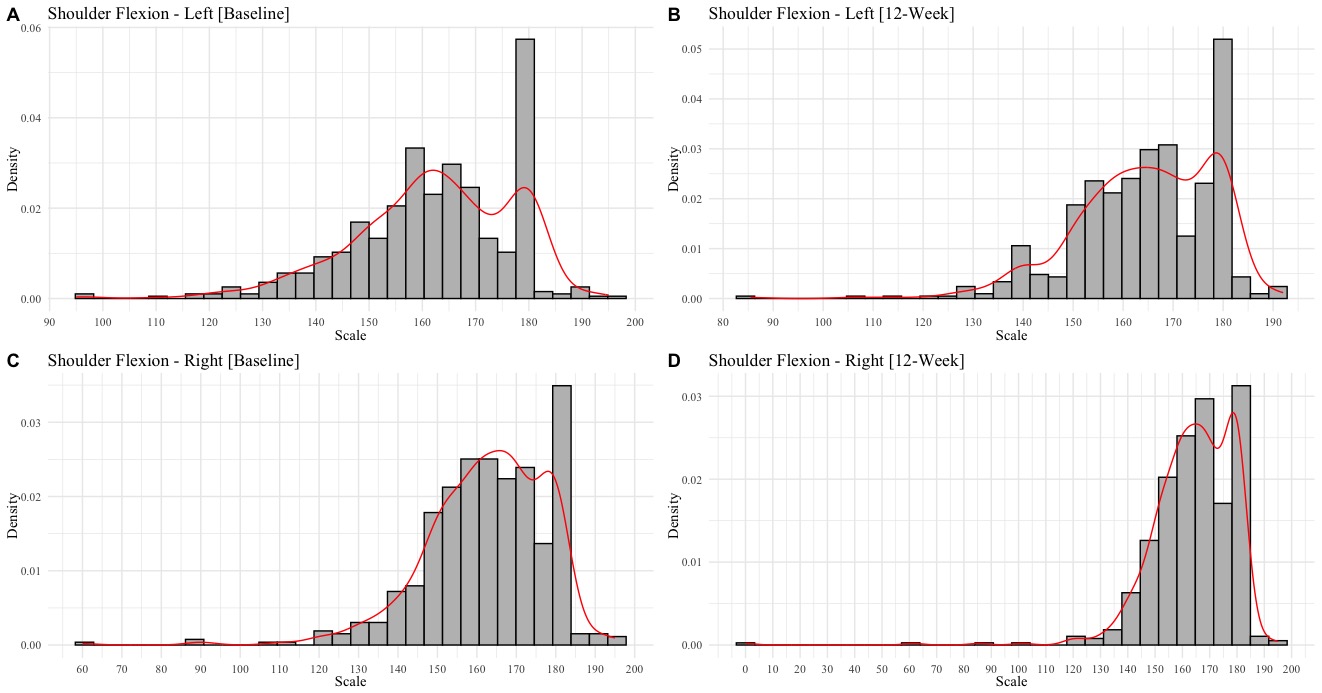


Supplemental Figure 3 A-D. Baseline and 12-Week Lower Body Flexibility


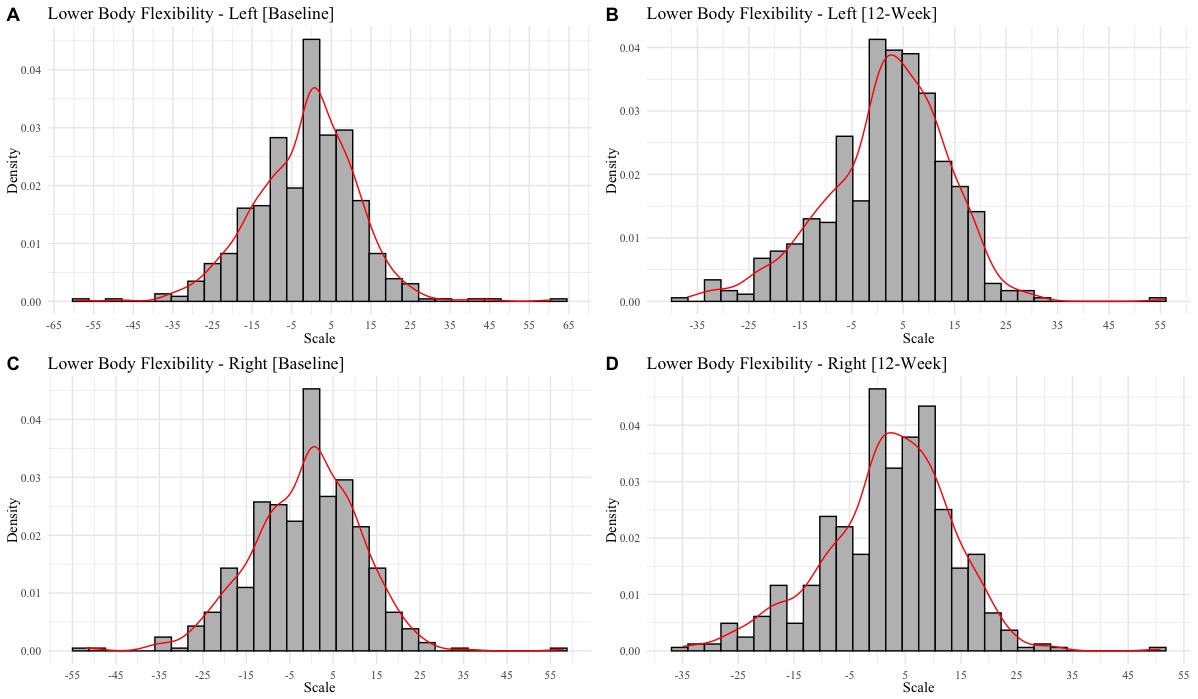


Supplemental Figure 4 A-D. Baseline and 12-Week Single Leg Stance


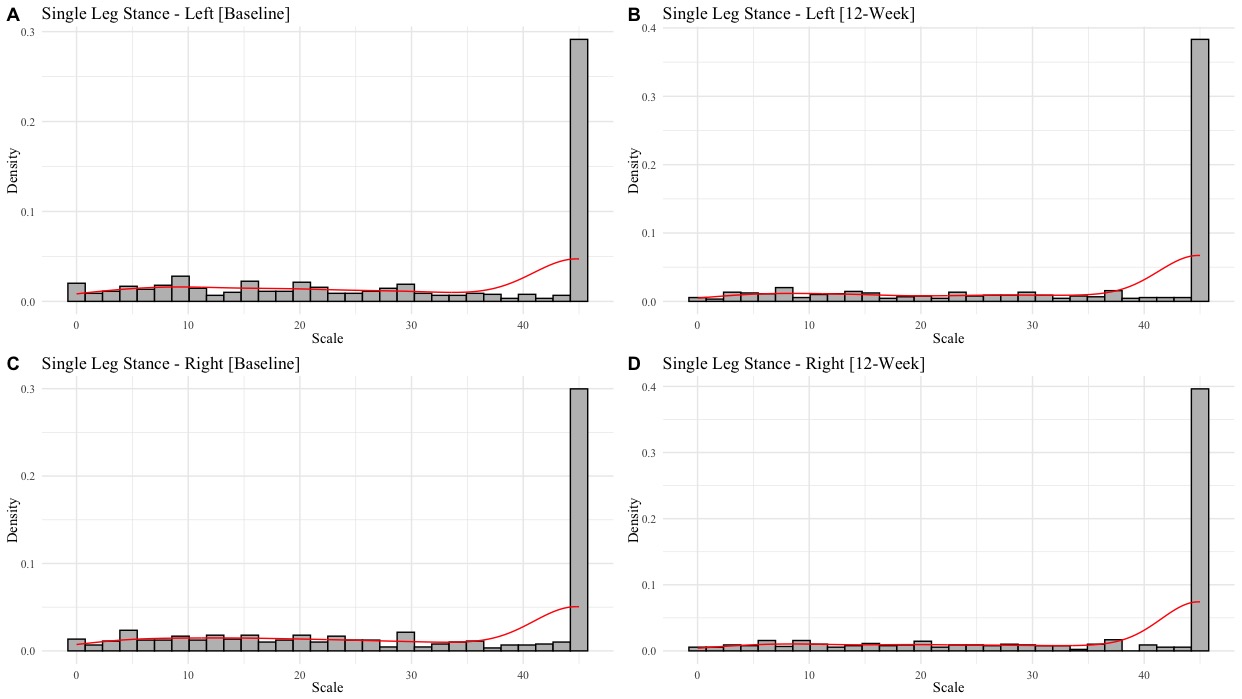


Supplemental Figure 5 A-D. Baseline and 12-Week 30-second Sit to Stand / 2-Minute Step Test


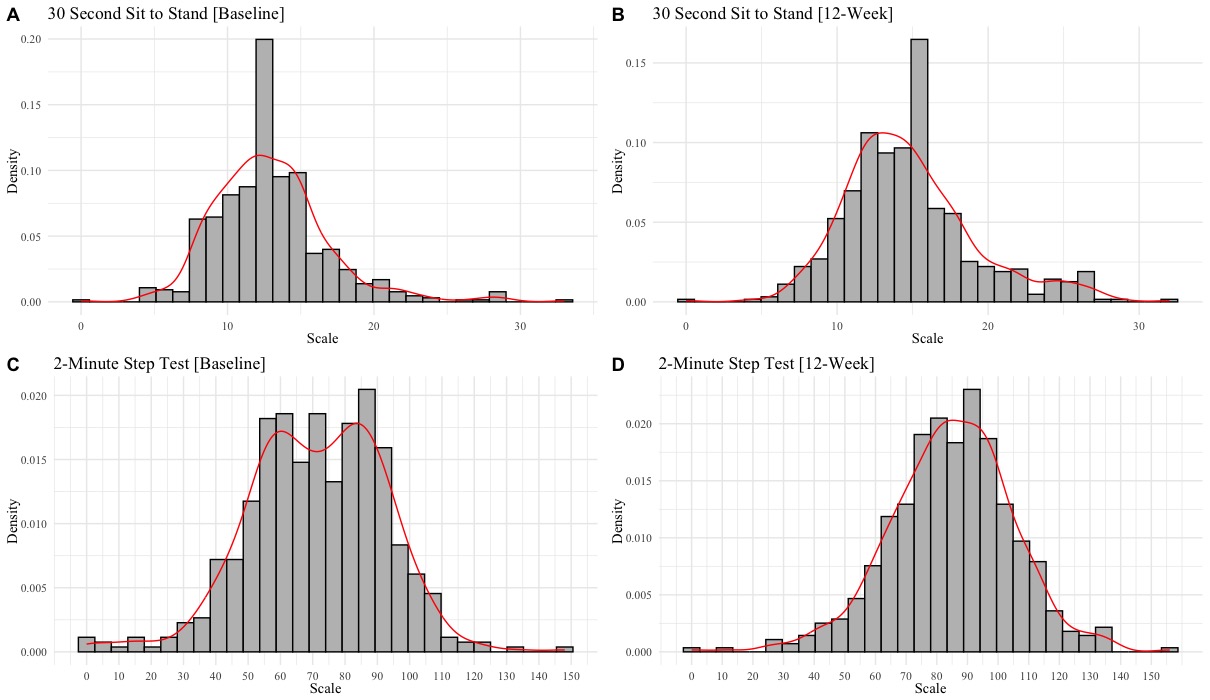

Supplement: Multimedia component 1 [file mmc1.docx]
